# The Psychological Impact of Slaughterhouse Employment: A Systematic Literature Review

**DOI:** 10.1177/15248380211030243

**Published:** 2021-07-07

**Authors:** Jessica Slade, Emma Alleyne

**Affiliations:** 1 School of Psychology, Keynes College, 2240 University of Kent, Canterbury, UK

**Keywords:** slaughterhouse worker, mental health, depression, anxiety, crime, coping mechanisms

## Abstract

The role of a slaughterhouse worker (SHW) involves the authorized killing of living beings, yet there is limited understanding of the consequences this behavior has on their well-being. The purpose of this systematic review is to collate and evaluate the current literature on the psychological impact of slaughterhouse employment. Fourteen studies met the specific a priori inclusion criteria. The findings from this review were demarcated by the focus of studies: (1) the prevalence of mental health disorders, (2) the types of coping mechanisms used, and (3) the link between slaughterhouse employment and crime perpetration. It was found that SHWs have a higher prevalence rate of mental health issues, in particular depression and anxiety, in addition to violence-supportive attitudes. Furthermore, the workers employ a variety of both adaptive and maladaptive strategies to cope with the workplace environment and associated stressors. Finally, there is some evidence that slaughterhouse work is associated with increased crime levels. The research reviewed has shown a link between slaughterhouse work and antisocial behavior generally and sexual offending specifically. There was no support for such an association with violent crimes, however. Based on existing research, we suggest future directions for research (i.e., applying more methodological rigor) but highlight key findings for practitioners and policymakers that warrant attention.

There are specific types of employment that require the authorized killing of living beings. Given the traumatic nature of this work, there has been research investigating the psychological impact, but only in a subset of professions (e.g., war veterans [MacNair, 2002], veterinarians, and researchers who conduct experiments on animals [Bennett & Rohlf, 2005]). However, very little is known about the consequences of working in slaughterhouses (also known as abattoirs). Slaughterhouse workers (SHWs) are involved in the deaths of more than 70 billion animals each year worldwide (Sanders, 2018). In order to meet market demand, the meat industry employs a workforce of approximately 75,000 people (British Meat Processors Association, 2019) in approximately 250 slaughterhouses in the United Kingdom (Department for Environment Food & Rural Affairs, 2019), with equivalent numbers in the United States (United States Department of Agriculture, 2020). Furthermore, statistics show that the majority of these employees have limited educational attainment and come from a low socioeconomic background (Victor & Barnard, 2016), with migrants making up 70% of the workforce in the United Kingdom (British Meat Processors Association, 2019).

There has been increased media coverage of the slaughterhouse industry as a result of the dissemination of online videos showing slaughterhouse staff abusing animals. Examples include using animals as a surface to extinguish cigarettes, decapitating animals and ridiculing their dismembered bodies, and inflicting abuse on animals as a form of game playing and entertainment (Animal Aid, 2015; Nagesh, 2017). In the United Kingdom, these videos prompted a change in legislation, whereby slaughterhouse establishments were required to install closed-circuit television (CCTV) to act as a deterrence, and if needed, to aid investigations (Embury-Dennis, 2018). However, animals are not the only victims of the slaughterhouse industry. Modern-day slaughterhouses prosper as a result of the industrialization of the production line (Hendrix & Brooks Dollar, 2017). Consequently, this puts immense pressure on the workers to keep up with such high demand (Dillard, 2008) resulting in violations of workplace policies (e.g., SHWs being denied bathroom breaks—Oxfam America, 2016; drug use to meet high production line demand—Hendrix & Brooks Dollar, 2017). Employment statistics, in addition to reports of high turnover (Fitzgerald, 2010), underline the need to better understand both short-term and longer-term psychological effects of working in such environments. Therefore, in the first instance, a consolidation of existing research findings, in the form of a systematic review, gives a springboard to build an evidence base that can inform practice and policy.

Before we embark on this review, we define a “slaughterhouse worker” to be an individual who works in a facility that kills and processes farmed animals for the consumption of meat. In the context of this form of employment, SHWs are exposed to serious risk of injury (Leibler & Perry, 2016), with amputations occurring, on average, twice per week in the United States (Wasley et al., 2018). Risk of injury is often attributed to the poor working conditions within slaughterhouses. For example, SHWs are often asked to work long shifts in cold, damp, and noisy environments (Campbell, 1999; Harmse et al., 2016; Human Rights Watch, 2004), with inadequate hygiene facilities (Cook et al., 2017). Furthermore, it has been argued that facilitating or observing the cutting, skinning, and boiling of conscious or unconscious animals can cause psychological distress (i.e., cognitive dissonance) on the workers (Eisnitz, 1997; Hendrix & Brooks Dollar, 2017). For example, there is a growing body of evidence that SHWs exhibit symptoms of post-traumatic stress disorder (PTSD) warranting clinical attention (Beirne, 2004). This has been further characterized as perpetration-induced traumatic stress, which is a form of PTSD where the person is involved (or believes they are involved) in creating the traumatic situation (MacNair, 2002). The resulting symptomatology—such as substance abuse, anxiety, nightmares, and depression—is debilitating. Nonetheless, the psychopathological consequences typically result in one of two outcomes. SHWs often attempt to attenuate the cognitive dissonance using maladaptive regulatory strategies (e.g., substance abuse, ruminative thinking) to enable them to perform their duties (Dillard, 2008; Niven et al., 2012). Alternatively, if the dissonance and psychological effects overcome coping strategies, SHWs come to the attention of mental health services (e.g., psychiatric inpatient services; Newkey-Burden, 2020).

The state of the literature on the psychological effects of slaughterhouse employment currently lacks a framework to point toward that outlines meaningful (theoretical and practical) assertions regarding the underlying mechanisms that facilitate poor mental health outcomes for the workers. This systematic review is timely because it gives the opportunity to take stock of the existing evidence and conceptualize research directions moving forward. Therefore, in an effort to orient researchers and identify gaps for future study, the purpose of this systematic review is to consolidate, synthesize, and evaluate the current literature on the psychological effects of working in slaughterhouses. Considering the findings gleaned from the existing body of research, we will also outline a framework for future research to further evidence the processes and mechanisms between workplace-facilitated trauma and its psychopathological consequences.

## Method

### Inclusion Criteria

The studies selected for inclusion criteria were those that examined any psychological aspect of slaughterhouse employment. Psychological effects were conceptualized as relating to any aspect of mental health, social and cognitive domains, and interpersonal relationships. The focus of the selected studies was purposely kept broad due to the scarcity of research. In order to be selected for final inclusion, studies were required to meet the following set of a priori criteria: (1) the focus of the study was to examine any of the psychological effects described previously, (2) written in (or translated to) English, (3) the article presented an empirical (quantitative or qualitative) study, rather than a review or theoretical argument, to enable sufficient quality appraisals. In addition to the inclusion criteria, the literature search was designed to capture both peer-reviewed and unpublished research to avoid publication bias (Trespidi et al., 2011).

### Document Search and Extraction

This review was guided by the Preferred Reporting Items for Systematic Reviews and Meta-Analysis (PRISMA) statement for reporting (Moher et al., 2009). A literature search was conducted across the following databases: Academic search complete, PsychArticles, PsychInfo, Scopus, and ProQuest Global Thesis Repository. The keywords used in the searches included slaughterhouse worker and “meatpacking worker.”

**Figure 1.**
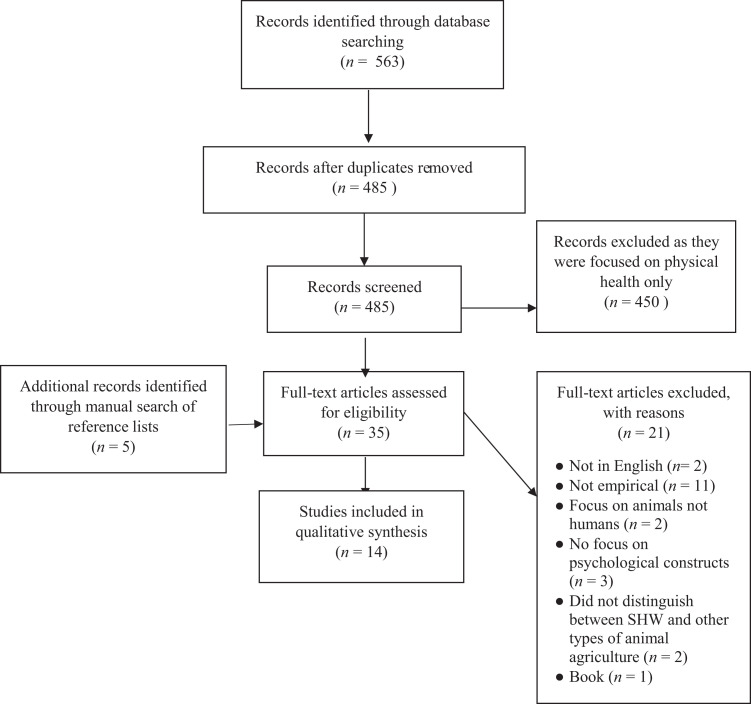
Flowchart of the literature search and study selection process.

The initial search generated 563 articles, with 485 remaining after duplicates were removed. After the titles and abstracts were examined against the a priori inclusion criteria, there were 30 remaining full-text manuscripts. Five additional journal articles were identified from the reference list of the 30 articles. No further articles were identified through contact with experts. Fourteen full-text articles met the inclusion criteria and were included in the review (see Figure 1 for study selection flowchart).

### Quality Appraisal

Two appraisal tools were used to provide a systematic method of assessing the quality of the studies. Qualitative papers were assessed using the Critical Appraisal Skills Programme (2016). Quantitative papers were assessed using the Quality Assessment Tool for Quantitative Studies (Thomas et al., 1998).

## Results

### Samples and Recruitment

Table 1 shows the details of the 14 studies used in this review. Half of the studies recruited participants from the United States (*n* = 7, 50%), the others recruited participants from the following countries: Australia, Brazil, Denmark, Ireland, South Africa, and Turkey. For the studies that examined SHWs (*n* = 12), there was a large variation in sample size, with a mean sample of 506 SHWs (minimum = 13, maximum = 4,407). Two studies used the same sample; that is, the study conducted by Horton and Lipscomb (2011) was a longitudinal analysis of Lipscomb and colleagues’ (2007) original study. The review included all-female studies (*n* = 2, 14%), all-male studies (*n* = 4, 29%), and mixed gender studies (*n* = 6, 43%). All of the studies used adult samples who were recruited through the following methods: internally (*n* = 2), placing adverts inside the slaughterhouse (*n* = 2), using community workers to circumvent the need to involve their employers (*n* = 2), national cohort (*n* = 2), snowballing techniques through personal connections (*n* = 1), and two papers did not specify. Three studies did not recruit participants: Two used secondary data and one used participant observation.

**Table 1.** Details of Studies Included in the Systematic Review.

**Authors**	**Type**	**Sample Characteristics** (sample size, demographics)	**Study Focus and Design** (quantitative/qualitative, study focus, analysis)	**Key Findings**
Baran et al. (2016)	Peer-reviewed article	*N* = 58 slaughterhouse workers (SHW) Location: Denmark Demographics: Mage = n/a Ethnicity = n/a Gender = n/a (demographic breakdown is only given for all occupations)	Quantitative design: survey Utilized dirty work theory to examine SHWs psychological well-being. Comparison between SHWs and 44 other professions.	SHWs have lower physical and psychological well-being (low self-esteem, lack of purpose, lack of personal development) compared with other professions, including controls matched on levels of prestige and dirtiness.
Emhan et al. (2012)	Peer-reviewed article	*N* = 43 SHW Location: Turkey Demographics: Mage = n/a Ethnicity = n/a Gender = all male	Quantitative design: survey Psychological symptom profiles of SHW compared with butchers and office workers.	SHWs had higher levels of distress and psychological issues compared with butchers for somatization, anxiety, anger hostility, and psychoticism. There was no significant difference between them on obsessive-compulsiveness, interpersonal sensitivity, depression, phobic anxiety, paranoia. All measures were significantly higher than office workers.
Fitzgerald et al. (2009)	Peer-reviewed article	*N* = 581 nonmetropolitan counties Location: United States	Quantitative: crime survey (secondary source) The link between crime rates and slaughterhouse employment	Slaughterhouse employment was associated with an increase in total arrests and reports, rape arrests, sexual offenses. Found no link between crimes against the family or violent crimes (after 1997).
Horton and Lipscomb (2011)	Peer-reviewed article	*N* = 296 SHW Location: United States Demographics: Mage = 33 Ethnicity = 99% Black Gender = all female	A longitudinal analysis of Lipscomb et al. (2007); tracking the prevalence of depressive symptoms over 5 years.	Depression rates were 46% at baseline interview (103 participants); however, the depression rates decreased over the 5 years (as did the number of participants). Average prevalence of depression was 32% across the 5 years.
Hutz et al. (2013)	Peer-reviewed article	*N* = 951 SHW Location: South Brazil Demographics: Mage = 32 Ethnicity = n/a Gender = mostly female (63.5%)	Mental illness due to adverse (stressful) working conditions Comparison between SHWs, university workers and students Quantitative.	SHWs had higher levels of vulnerability, psychological “disadjustment,” anxiety, and depression compared with both controls. Within SHW—those involved in cutting processes have much higher rates.
Jacques (2015)	Peer-reviewed article	*N* = 248 non-Metropolitan counties Location: United States	Quantitative: crime survey (secondary source) The link between crime rates and slaughterhouse employment when controlling for social disorganization variables.	Increase in total arrests (22%), rape (166%), offenses against the family (90%) but nonsignificant results for violent and murder offenses. No indication of whether the workers actually committed the crime though.
Kristensen (1991)	Peer-reviewed article	*N* = 4407 SHW Location: Denmark Demographics: Mage = Ethnicity = n/a Gender = mostly male (78%)	Quantitative: survey Primary focus was on their physical health but also examined stress symptoms.	Findings demonstrated that half the sample suffered from stress symptoms (common stress, nervousness, mental instability, anxiety, and sleeplessness).
Lander et al. (2016)	Peer-reviewed article	*N* = 268 SHW Location: United States Demographics: Mage = 39 Ethnicity = majority non-Hispanic Gender = mostly male (64%)	Quantitative: survey Whether depression was a risk for future injury.	13.8% screened positive for depressive symptoms in the last 6 months, compared with 3.4% in general public.
Leibler et al. (2017)	Peer-reviewed article	*N* = 137 unionized SHW Location: United states Demographics: Mage = 44 Ethnicity = 92% Hispanic Gender = mostly male (55%)	Quantitative: Interviews and survey (Kessler-6) Examine the prevalence of serious psychological distress (SPD; nonspecific anxiety)	SPD was 4.4% in slaughterhouses compared with 3.6% in general population in the last 6 months. 81% reported no distress Those on the kill floor experienced more distress compared with those on the cut floor. Ethnicity was a significant predictor of SPD; Non-Hispanic White workers were six times more likely to report SPD. However, the authors argued that this is a result of being the minority group.
Lipscomb et al. (2007)	Peer-reviewed article	*N* = 291 SHW Location: United States Demographics: Mage = Ethnicity = 98.5% Black Gender = all female Multiple jobs	Quantitative: surveys Prevalence of depression in female poultry processers compared with locals and examine which factors are associated with depression.	Prevalence of depressive symptoms was 48% compared with 20% of working women. After adjusting for socioeconomic variables, the poultry workers’ depressive symptoms were still 80% higher. Prevalence of severe depression was 550% higher than controls.
McLoughlin (2018)	Peer-reviewed article	*N* = 16 SHW Location: Ireland Demographics: Mage = n/a Ethnicity = n/a Gender = mostly male (87.5%) Kill floor only	Qualitative: Interviews and participant observation. Emic phenomenological emotionography.	Findings indicated that the hegemonic masculine ideals are the basis of a “good SHW” and thus workers must deny, repress, or diminish any emotions they feel.
Richards et al. (2013)	Peer-reviewed article	*N* = 26 SHW Location: Australia Demographics: Mage = 36 Ethnicity = n/a Gender = mostly male (54%)	Quantitative: survey Examine the attitudes toward animals and propensity for aggression	SHWs had a substantially higher propensity for aggression compared with farmers, particularly within physical aggression and hostility subscales (similar to incarcerated populations). Female SHWs, in particular, had lower attitudes toward animals and a higher propensity for aggression than males.
Thompson (1983)	Peer-reviewed article	*N* = 350 SHW Location: United States Demographics: Mage = n/a Ethnicity = 2/3 White Gender = male	Qualitative: 9-week participant observation. To examine how SHWs cope with the strains of their work and maintain a sense of self-worth	SHWs must struggle with the fear of physical danger, monotony (which sometimes causes injury and the dehumanization of becoming part of “the machine.” Suggested that workers cope by sabotaging their own/others’ work, as it allows them to express their individuality.
Victor and Barnard (2016)	Peer-reviewed article	*N* = 13 SHW Location: South Africa Demographics: Mage = mostly 30–40 Ethnicity = n/a Gender = all male	Qualitative: phenomenological To examine the well-being of SHWs and understand the process of becoming a slaughterer.	Four themes: becoming a slaughterer (experiencing the mental trauma of the first kill and experiencing recurring dreams and nightmares), (mal)adjusting to slaughter work (heightened emotive responses, personality changes), coping with and maintaining the work (presenting psychological defenses, finding strength and meaning, displaying constructive and destructive coping tactics), and living with psychosocial consequences of being a slaughterer (work–life spillover, experiencing social detachment, and isolation).

The majority of studies examined slaughterhouses that processed cattle (*n* = 5, 36%), whereas the others were poultry (*n* = 3, 21%) and pork (*n* = 1, 7%) establishments. Fitzgerald et al. (2009) used both cattle and pork and excluded poultry. Four papers did not specify (29%) which animals were processed. Furthermore, seven papers (50%) specified which role the workers had in the slaughterhouse process, of which three focused exclusively on workers on the kill floor (21%) and the rest compared the kill floor to other positions.

### Study Focus and Design

Most of the studies (*n* = 8, 57%) focused on the prevalence of mental health issues within slaughterhouse employees, four examined how SHWs cope with aspects of their employment (29%), and two studies examined the link between slaughterhouse employment and crime (14%). Within those which focused on mental health, one paper was actually focused on the physical health of its participants but examined depression as a risk factor for future injury (7%; Lander et al., 2016). Seven articles (50%) shared the hypothesis that the intentional killing or dismemberment of animals would have an impact on their well-being, in particular: general well-being (Baran et al., 2016), or linked with depression (Emhan et al., 2012; Horton & Lipscomb, 2011; Hutz et al., 2013; Lipscomb et al., 2007), anxiety (Emhan et al., 2012; Hutz et al., 2013; Leibler et al., 2017), and psychosis (Emhan et al., 2012). Two studies examined aspects of SHWs’ mental health which may have an impact on interpersonal relations such as anger and hostility (Emhan et al., 2012; Richards et al., 2013).

Among the studies that focused on the prevalence of mental health issues, all were quantitative, utilizing self-report questionnaire measures, with acceptable or above Cronbach’s αs, and had a control or reference group. Two articles solely compared their findings against the national average (Lander et al., 2016; Leibler et al., 2017). Lipscomb and colleagues (2007) compared SHWs to individuals from the same community. The other articles (*n* = 4, 29%) used two control groups: one whose participants were theoretically matched to SHWs and one nonmatched (typically individuals from the same community). The matched control groups depended on the theory of the researcher. One article (Baran et al., 2016) came from a dirty work perspective and matched SHWs with jobs rated similarly on levels of prestige and “dirtiness” (janitors and homecare workers) by experts in dirty work theory and then compared them with 44 other professions. Hutz and colleagues (2013) compared SHWs to university staff as matched for stressful environments and then used university students as a control against both groups. Two articles compared SHWs with jobs relating to animals: butchers (Emhan et al., 2012) and farmers (Richards et al., 2013). The majority (*n* = 4) used a form of regression to analyze their data. The rest used one of the following methods: *t* test, analysis of variance, and mixed-model design.

The next key theme generated from the studies focused on how SHWs coped with the demands of their work (*n* = 4). However, the studies had variations on how they defined what SHWs were coping against. Kristensen (1991) focused on the risk of physical injury. Thompson (1983) focused on how SHWs cope with the monotonous but physically demanding and dangerous nature of such work. McLoughlin (2018) and Victor and Barnard (2016) focused on how workers coped with the psychological toll of slaughtering animals. One study (Kristensen, 1991) used self-report questionnaires. The others utilized a qualitative design: that is, Thompson (1983) used participant observation, Victor and Barnard (2016) used unstructured interviews, and McLoughlin (2018) used a combination of the two. Both interview studies were conducted from a phenomenological perspective, with McLoughlin (2018) utilizing the participant observation to give an emic perspective.

The final theme from the research examined the relationship between slaughterhouse employment and associated crime in the community (*n* = 2). Both articles had the same hypothesis: slaughterhouse employment was associated with an increase in crime. Rather than examining SHWs themselves, both articles examined the link between the presence of a slaughterhouse and associated crime in a US non-Metropolitan county. The studies had two different independent variables: the number of employees (Fitzgerald et al., 2009) and the number of slaughterhouse establishments (Jacques, 2015). Fitzgerald and colleagues (2009) operationalized crime as total arrests and reported crimes, and Jacques (2015) only utilized total arrests. They looked for the same types of crimes: total, family, assault, violent crimes, murder, rape, and other sexual offenses. They both controlled for variables that are typically associated with crime such as demographics and unemployment rate. Additionally, Fitzgerald and colleagues (2009) further controlled for the poverty rate and migration, and Jacques (2015) controlled for female-headed households and population density. Both justified their control variables from the literature, stemming from social disorganization and crime theory. Furthermore, Fitzgerald and colleagues (2009) ran further analyses to investigate whether similar jobs (characterized by high levels of immigrant workers, low pay, routinized labor, and dangerous conditions) differed from slaughterhouse employment on their associated crime rates. Both reports used a negative binomial regression analysis, and Fitzgerald and colleagues (2009) also used an Ordinary Least Squares (OLS) regression for total arrests and total reports of crime.

### Key Findings

As mentioned previously, the 14 studies included in this systematic review examined the psychological effects of slaughterhouse employment. The key findings of these studies will be presented in three sections: the prevalence of mental health issues, coping mechanisms, and the link with crime perpetration.

#### Prevalence of mental health issues

All of the studies concluded that SHWs have lower levels of psychological well-being compared with their respective control groups. The qualitative work conducted by Victor and Barnard (2016) found that South African SHWs reported suffering from the following psychological issues at the beginning of their employment as a consequence of their first kill: trauma, intense shock, paranoia, fear, anxiety, guilt, and shame. These findings were supported by studies employing quantitative methods. Kristensen (1991) found that half of their sample had high levels of stress-related symptoms. Furthermore, Baran and colleagues (2016) concluded that SHWs have significantly lower levels of psychological well-being compared with other professions (44 types), as they have lower levels of self-esteem, purpose, and personal development. The effect size was small but significant. The authors also conducted separate analyses where they identified similarly rated “dirty work” professions (professions that received virtually the same expert ratings on prestige and dirtiness; i.e., janitors and home care workers) and compared them to the other professions to see if there were differences in their psychological well-being. They found that these nonslaughterhouse dirty work professions did not differ from the other professions on negative outcomes. This suggests that such psychological consequences may be a distinct outcome of working in a slaughterhouse.

For depression, significant differences were found in all comparative studies (i.e., SHWs indicated higher levels of depression than the comparison group; Hutz et al., 2013; Lander et al., 2016; Lipscomb et al., 2007), with the exception of Emhan and colleagues (2012). They found that SHWs had significantly higher levels of depression compared with office workers, but not butchers. The difference in depression rates differed from study to study, ranging from 10% to 50%. Lander and colleagues (2016) found that the prevalence of depression was four times higher than the national average. Lipscomb and colleagues (2007) found that rates of severe depression were more than five times higher than their reference group, controlling for gender and socioeconomic variables.

Similar findings were reported for anxiety, with SHWs having a higher prevalence compared with other professions (Emhan et al., 2012; Hutz et al., 2013) and the general public (Leibler et al., 2017). One study examined the relationship between ethnicity and anxiety, finding that non-Hispanic Whites were six times more likely to experience serious psychological distress. However, they attributed the finding to anxiety caused by their minority ethnicity status within the workplace (Leibler et al., 2017). Emhan and colleagues (2012) found that SHWs also had significantly higher levels of psychoticism, somatization, anger, and hostility compared with butchers and office workers. Similarly, Richards and colleagues (2013) found that SHWs had a higher propensity for aggression compared with the public and farmers, on all aspects of aggression (physical aggression, anger, and hostility) except verbal aggression, which was approaching significance. Interestingly, the women in their sample had a significantly higher propensity for aggression scores than the men.

Staff with the job role involving the slaughtering process itself were found to exhibit higher rates of mental health problems. Hutz and colleagues (2013) found that workers in the cutting sector had significantly higher prevalence rates of depression and anxiety compared with other roles in the slaughterhouse. Similarly, Richards and colleagues (2013) found that a propensity for aggression was also related to job roles, with the highest scores of aggression being associated with working in the “load outs” (i.e., handling the carcasses), followed by working on the kill floor, then the other roles. However, it is worth noting that the small sample size could have impacted on findings.

#### Coping mechanisms

Each study identified different types of coping mechanisms. Kristensen (1991) originally theorized that workers take days off to cope with the demands of the job. He argued that “sick days” were the result of workers being incapable of coping with the lack of breaks and therefore needed extended lengths of time to recuperate. When examining his data, he found that half of the participants had elevated levels of stress, however, the primary reason for taking time off work was to cope with physical injuries rather than psychological strain. In related work, Thompson (1983) found that SHWs struggled with the fear of physical harm. This fear was amplified by the monotony of their work. Workers often daydreamed to escape boredom, which resulted in an increase in injuries. There were also issues of victim blaming. The workers would attribute blame to the colleague who got injured rather than justify the accident as a result of workplace conditions. Furthermore, Thompson (1983) argued that the most psychologically impactful aspect of the work was the dehumanization, whereby workers described their role as part of a machine and thus easily replaceable. This was amplified by the social environment, as the workers were unable to interact with each other due to the excessive noise of the machinery and their fixed position on the production line. A consequence of the monotonous, machine-like environment was the workers’ use of sabotage as a coping mechanism. That is, causing disruption was a symbolic method of expression of individuality and self-worth (Thompson, 1983).

Two studies examined how workers coped with the specific act of slaughtering of animals. McLoughlin (2018) posited that SHWs needed to conform to hegemonic masculinity in order to successfully complete their work. The reasoning underpinning this conformity was that emotions impeded their work, caused internal conflict, and lowered their status in the eyes of their peers. Thus, McLoughlin argued that workers deny, diminish, or repress their emotions as a form of a self-regulating coping mechanism. Victor and Barnard (2016) conceptualized the process of coping with slaughterhouse work into four stages. First, workers experience the identity shift of becoming a slaughterer, which is characterized by the mental trauma of their first kill and the, sometimes recurring, nightmares. Second, they (mal)adjust to their work, with some workers reporting heightened affective responses (e.g., guilt and shame) and personality changes (e.g., becoming more aggressive). Third, they begin to display (mal)adaptive coping mechanisms to enable them to continue working. Some participants found helpful ways to cope, such as relying on support from their family, community, or religion. However, others employed maladaptive coping mechanisms, including emotional detachment (akin to what McLoughlin [2018] theorized), self-medicating with drugs and alcohol, or resorting to violence. Workers also described the psychosocial consequences of the “job-home spillover,” such as social detachment due to exhaustion, or even the perpetration of violence, typically in a domestic context.

#### Crime link

Two articles quantitatively examined the work spillover effect described in Victor and Barnard’s (2016) study. Fitzgerald and colleagues (2009) examined crime reports from 1994 to 2002, whereas Jacques (2015) used data from 2000. Both articles found that slaughterhouse employment was associated with a significant increase in total arrests and arrests for sexual offending (i.e., rape) across all time periods, controlling for demographic and socioeconomic factors. Interestingly, Fitzgerald and colleagues (2009) found a significant negative effect on the number of rapes being reported. Contrary to their hypothesis, they both found no significant relationship between slaughterhouse employment and violent crime (i.e., aggravated assault and murder) during the same time period (from 1997 onward). However, Fitzgerald and colleagues found a significant positive relationship between 1994 and 1997. The studies had conflicting results for sexual offenses (not including rape) and crimes against the family.

## Discussion

The purpose of this systematic review was to consolidate and synthesize the empirical research that examines the psychological impact of slaughterhouse employment. In summary, 14 studies met the inclusion criteria for this systematic review. Upon examination, the studies were delineated by study focus. Eight studies examined the self-reported prevalence of mental health issues in SHWs, four studies focused on the types of coping mechanisms used by SHWs, and two studies examined the link between slaughterhouse employment and crime.

There is evidence that slaughterhouse employment is associated with lower levels of psychological well-being. SHWs have described suffering from trauma, intense shock, paranoia, anxiety, guilt and shame (Victor & Barnard, 2016), and stress (Kristensen, 1991). There was evidence of higher rates of depression (Emhan et al., 2012; Horton & Lipscomb, 2011; Hutz et al., 2013; Lander et al., 2016; Lipscomb et al., 2007), anxiety (Emhan et al., 2012; Hutz et al., 2013; Leibler et al., 2017), psychosis (Emhan et al., 2012), and feelings of lower self-worth at work (Baran et al., 2016). Of particular note was that the symptomatology appeared to vary by job role. Employees working directly with the animals (e.g., on the kill floor or handling the carcasses) were those who showed the highest prevalence rates of aggression, anxiety, and depression (Hutz et al., 2013; Richards et al., 2013).

Given the psychological and psychopathological demands of slaughterhouse employment, the workers engage in a range of coping strategies. Some of the strategies are helpful and adaptive, such as taking days off work (Kristensen, 1991), and relying on prosocial forms of support (e.g., family or religion; Thompson, 1983). However, oftentimes, the workers employ strategies that are maladaptive, such as repressing difficult emotions (McLoughlin, 2018; Victor & Barnard, 2016), sabotaging their working environment as a form of expression (Thompson, 1983), using illicit substances, and/or engaging in interpersonal violence (Victor & Barnard, 2016). Therefore, it is unsurprising that crime statistics indicate a positive association between the presence of slaughterhouse establishments and crime arrests generally and rape arrests specifically (Fitzgerald et al., 2009; Jacques, 2015).

### Limitations

The research reviewed was not without its limitations, and these limitations constrained the bearing of some of the conclusions. In particular, there were variations in the rigor of the research designs. For example, the use of control groups to evidence differences in mental health symptoms and diagnoses was useful to contextualize the vulnerability of SHWs. However, some comparisons were more informative than others. It is only possible to conclude that there was something unique about slaughterhouse employment that was driving the prevalence of mental health issues if the groups only differ on one factor. If multiple differences were found, then conclusions cannot be confidently drawn as to which of the factors may be driving the effects (i.e., varying prevalence rates). Hence, these conclusions must be considered with caution. For example, two articles (Lander et al., 2016; Leibler et al., 2017) compared mental health prevalence rates against the national average. Although this provided a normative baseline, this may be a questionable comparison to make since there is such a large within-group variation of depression rates across the United States, and thus a large number of confounding variables. Lipscomb and colleagues (2007) made a more informative comparison by recruiting a control group from the same community but had not worked in the slaughterhouse for at least 5 years and were matched by age, gender, and controlled for socioeconomic variables, thus reducing the number of confounding variables. They found that simply working in the slaughterhouse, compared with a similar individual (in relation to their demographics) from the same town, is still likely to result in a higher prevalence rate of depression.

Other studies used two comparison groups in order to further reduce confounds: a theoretically matched control and then a dissimilar group to compare against. These study designs, although more rigorous, do come with their own issues regarding the matched controls. The researchers argued that their theoretical controls enabled them to examine whether an aspect of slaughterhouse work (typically the slaughtering of animals) was markedly different from jobs that are similar on other variables. For example, two studies matched SHWs with other jobs which involved handling farmed animals (i.e., butchers [Emhan et al., 2012] and farmers [Richards et al., 2013]). Although these comparisons may make intuitive sense, since all of those professions are involved in the meat production process, they are markedly different from SHWs. Farmers work with live animals and raise/nurture them for slaughter, and butchers process the “stock” (i.e., the already slaughtered animals) and provide a service akin to retail work. Richard and colleagues’ (2013) research was able to identify that SHWs differ significantly on levels of aggression and hostility but was unable to infer which part of slaughterhouse employment causes these effects. Two studies attempted to isolate factors within slaughterhouse employment which they believed were causing the effects. Hutz and colleagues (2013) hypothesized that it was the stressful environment that decreased the workers’ psychological well-being, but that there was something unique to slaughterhouse employment over and above stressful conditions. Therefore, they used a control group of university staff, who they argued had equally stressful jobs. However, they did not provide any evidence for how they matched the two professions on stress levels. Baran and colleagues’ (2016) research stemmed from dirty work theory and thus matched SHWs with similarly “dirty” jobs. Unlike Hutz and colleagues (2013), they used independent experts in the field to rate 44 occupations on two key areas of dirty work (prestige and dirtiness), and then selected two professions that had similar mean scores to the ratings of SHWs. Thus, this matched comparison was achieved more rigorously and it was grounded in theory.

Importantly, these studies have highlighted associations between slaughterhouse employment and detrimental effects on mental health and behavior (i.e., criminal behavior), however, the research designs do not allow us to infer causality. There is a tendency to assume that slaughterhouse employment *causes* these poor outcomes. The data, so far, can neither confirm nor dispute this assumption. Theoretically speaking, there is room for counterarguments, one of which is the process of self-selection. That is, individuals with mental health difficulties and/or antisocial proclivities could choose this form of employment for a variety of reasons. Slaughterhouse employment is typically low-skilled, low-pay work. People who already have a criminal record will likely have limited employment opportunities available to them. Slaughterhouse establishments are also more likely to be located in low-income areas where mental health issues are more prevalent, resulting in this form of employment being one of the limited options available. Ultimately, there is insufficient evidence to substantiate whether slaughterhouse employment causes detrimental effects, or whether people with existing vulnerabilities are attracted to this form of employment.

What is abundantly clear from this review is that more research is needed. The limited number of studies is indicative of a wider issue. There are challenges to gaining access to recruit participants for a number of reasons. Some employers might be concerned that research would lead to significant policy (and financial) changes if workplace conditions are indeed found to cause psychological and physical harm. Other employers might be concerned that the research is underpinned by animal welfare motivations to cease their business practices. Essentially, their skepticism results in an unwillingness to allow access to researchers. Nonetheless, people who work in slaughterhouses appear to be particularly vulnerable regardless of whether this form of employment is the cause or another symptom, and we have a duty of care to conduct further research.

### Future Directions

Future research must first begin with “buy-in” from business allies (i.e., slaughterhouse employers) to work collaboratively in setting and carrying out a research agenda. Slaughterhouse employment is linked to psychosocial sequelae that impact surrounding communities. Current conditions are not sustainable, given the evidence for high turnover (i.e., Fitzgerald, 2010) and mental health needs of employees as discussed in this review. Therefore, a collaborative approach to this research can result in a better understanding of the problem and an evidence base to inform effective solutions.

With growing opportunities for research must come an improved, rigorous approach to the study designs. One of the research questions that need to be urgently addressed is whether slaughtering animals causes mental health issues and criminal behavior. The only way to answer this question is to conduct a longitudinal study that can demonstrate, over time, whether people who work in slaughterhouses have declining mental health and an increase in antisocial behavior. This research must also involve a matched control group of similar age, ethnicity, socioeconomic background, and location/neighborhood. Only then can we evidence cause and effect so that the appropriate interventions can be developed to target appropriately.

Finally, as the number and quality of studies grow, there will be an opportunity to conduct a meta-analysis across studies. This will enable us to establish within- and between-study similarities and differences that can inform larger scale policy developments to reduce physical and psychological harm to slaughterhouse employees.

## Conclusions

The findings of this review illustrate the scarcity of research on the psychological well-being of SHWs. The existing research evidences the relationship between this form of employment and negative psychological and behavioral outcomes, both at the individual level and for the broader society. Also, these findings have clear implications for mental health and community professionals who are in a position to address the negative consequences of this industry. However, much more theoretical and empirical work is needed to develop the evidence base for developing prevention and intervention strategies.

### Implications for Research, Policy, and Practice

#### Research

- Research is needed to explicate the underlying mechanisms and processes linking slaughterhouse employment and both psychological (i.e., mental health) and behavioral (i.e., antisocial behavior) outcomes.
- There is a critical need for research examining the psychological characteristics of individuals who seek employment in slaughterhouses and the longer-term effects of animal killing.

#### Policy

- Slaughterhouse employers should review the range of possible explanatory factors in this review for employee burnout, turnover, and other performance issues.
- Implementation of clinical supervision requirements for slaughterhouse employees would help in the early identification of psychological well-being issues. This would also protect against employee burnout, turnover, and associated performance issues.
- Independent inspections of slaughterhouse facilities should also include a review of employee support provision.

#### Practice

- This review offers an overview of potential treatment needs for practitioners (e.g., Criminal Justice System professionals, psychologists, occupational health practitioners).
- Protocols for clinical supervision in mental health settings will have transferrable content as a baseline. Further development and evaluation of protocols that are accessible to slaughterhouse establishments could lead to a reduction in the psychological and behavioral outcomes outlined in this review.
